# Analysis of 90-day risk factors for poor prognosis in patients with postoperative CT with hyperdense signs after thrombectomy for acute ischemic stroke

**DOI:** 10.3389/fneur.2025.1609722

**Published:** 2025-11-26

**Authors:** Zhen Zhang, Lirong Yi, Bo Xiang, Jie Zhou

**Affiliations:** Department of Radiology, The Affiliated Yongchuan Hospital of Chongqing Medical University, Chongqing, China

**Keywords:** mechanical thrombectomy, hyperdense signs, postoperative CT, acute ischemic stroke, risk factors, prognosis

## Abstract

**Objective:**

To investigate the risk factors associated with poor 90-day prognosis in patients exhibiting hyperdense signs on the initial CT scan after mechanical thrombectomy for acute ischemic stroke.

**Methods:**

We conducted a retrospective analysis of 96 patients with acute ischemic stroke who underwent mechanical thrombectomy at The Affiliated Yongchuan Hospital of Chongqing Medical University. The patients were divided into a training set (*n* = 55, from August 2020 to March 2022) and a validation set (*n* = 41, from November 2022 to December 2023). Based on the 90d mRS scores, patients were categorized into a good prognosis group (*n* = 46) and a poor prognosis group (*n* = 50). Clinical and imaging data were compared between the two groups. A prediction model was constructed using the logistic regression algorithm and validated using the temporal validation method. Receiver operating characteristic (ROC) curves were generated, and the area under the curve (AUC) was calculated to evaluate the discriminative ability of the model. The Hosmer–Lemeshow goodness-of-fit test was used to assess calibration, and decision curve analysis was performed to evaluate the clinical net benefit of each model across various threshold probabilities.

**Results:**

Logistic regression analysis identified the following risk factors for poor 90-day prognosis: D-dimer level, admission diastolic blood pressure, preoperative NIHSS score, vascular occlusion site, as well as the volume of hyperdense areas and the total volume of both hyperdense and hypodense areas on the first postoperative CT scan. Two models were developed: one based solely on an imaging indicator (total volume) and another incorporating combined indicators (total volume, D-dimer, admission diastolic blood pressure, preoperative NIHSS score, and vascular occlusion site). The combined-indicator model demonstrated superior performance. In the training set, it achieved an AUC of 0.886 (95% CI: 0.801–0.971, *p* < 0.001), accuracy of 0.818, sensitivity of 0.818, and specificity of 0.818. In the test set, the AUC was 0.848 (95% CI: 0.730–0.966, *p* < 0.001), with an accuracy of 0.707, sensitivity of 0.542, and specificity of 0.941. Decision curve analysis confirmed that two models maintained a positive clinical net benefit within a wide range of threshold probability values (10%–90%).

**Conclusion:**

Patients with hyperdense signs on the first postoperative CT scan exhibit distinct risk factors for poor 90-day prognosis. Combining imaging features with clinical indicators significantly improves the predictive value for 90-day outcome after mechanical thrombectomy.

## 1 Introduction

As a pivotal endovascular treatment for revascularizing occluded vessels, mechanical thrombectomy (MT) has become the first-line therapy for acute ischemic stroke (AIS), achieving high rates of successful recanalization and improved clinical outcomes (1–3). However, postoperative cerebral hemorrhage remains a major complication and a leading cause of poor prognosis after MT (4, 5). Non-contrast computed tomography (CT) is routinely used for postoperative assessment, including the detection of hemorrhage and evaluation of treatment efficacy. Postoperative hyperdense signs on CT may arise from contrast staining, hemorrhage, or a combination of both; however, visual interpretation alone often fails to reliably differentiate these entities (6). These hyperdense signs are observed in 31.2%–87.5% of patients, with approximately 46% progressing to hemorrhagic transformation, yet they exhibit low positive predictive value and specificity (7). Currently, short-interval follow-up imaging serves as the primary clinical method to distinguish contrast extravasation from true hemorrhage, relying on the dynamic changes: contrast media typically wash out rapidly, while hemorrhage persists or expands. This approach, however, delays definitive diagnosis and may impede timely clinical decision-making. Furthermore, repeated CT scans increase radiation exposure, underscoring the need for more immediate and reliable prognostic tools.

The presence of hyperdensity reflects underlying blood–brain barrier (BBB) disruption and vascular injury, which permit extravasation of contrast or blood into the brain parenchyma (8, 9). Additionally, hypodense regions often correspond to evolving ischemic edema or necrotic tissue, and their volumetric extent may also serve as an important indicator of tissue vulnerability and clinical outcome. The progression of hemorrhagic transformation can significantly worsen prognosis and offset the benefits of thrombectomy, highlighting the importance of early risk stratification. Although previous studies have reported inconsistent associations between hyperdense signs and clinical outcomes (10–12), most rely on qualitative assessment rather than quantitative volumetric analysis. There is a growing recognition that both hyperdense and hypodense volumes on early postoperative CT may offer predictive value, yet the integration of these imaging biomarkers into multivariable prognostic models remains underexplored.

Accordingly, the present study seeks to develop a logistic regression model that integrates quantitative volumetric measurements of both hyperdense and hypodense regions from the first postoperative CT scan with key clinical variables to predict 90-day functional outcomes after thrombectomy. We seek to develop an early predictive tool that facilitates individualized risk assessment and supports clinical decision-making immediately after thrombectomy.

## 2 Methods

### 2.1 Patients

**Figure 1:**
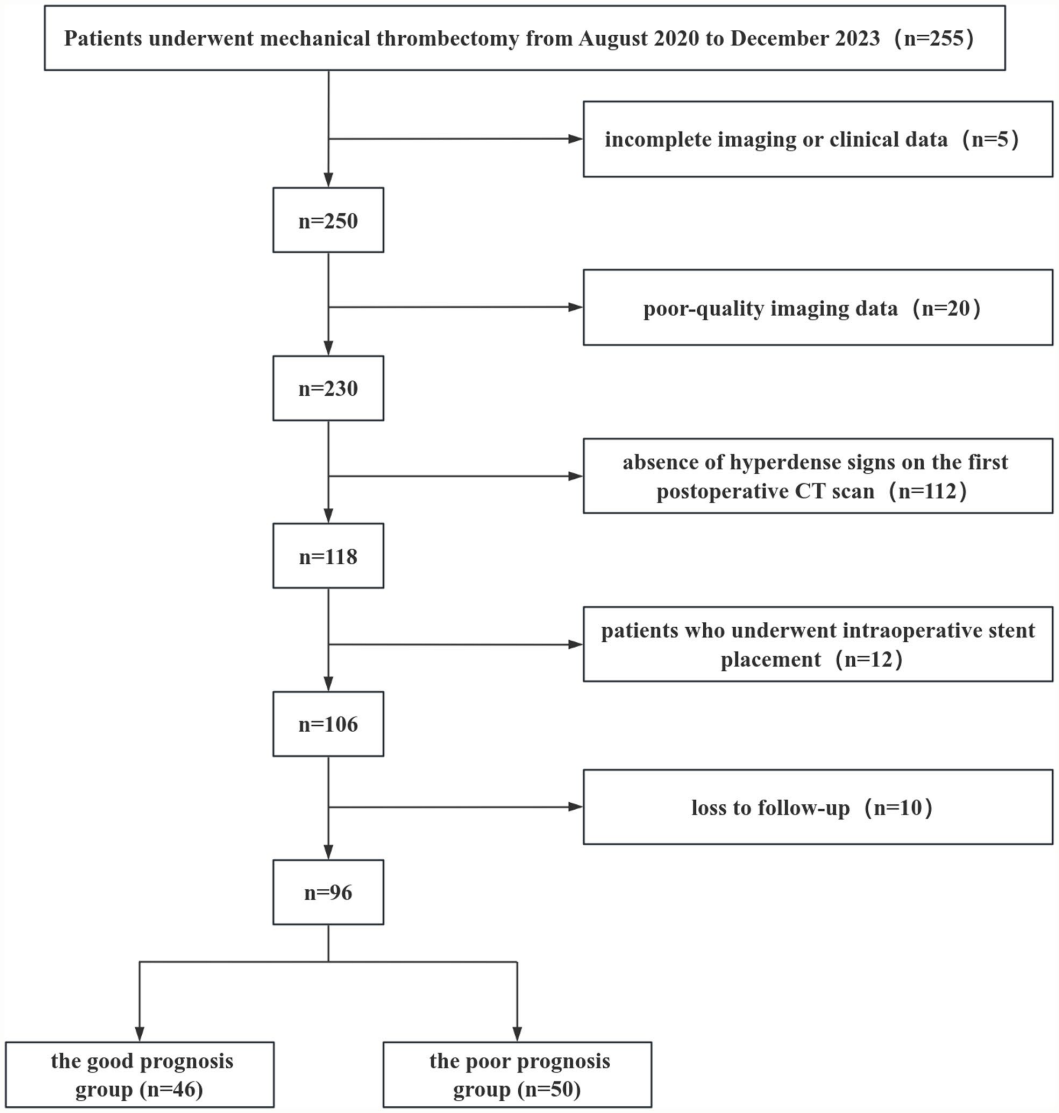
Flowchart of patients selection.

A retrospective study was conducted on 96 consecutive patients with acute ischemic stroke who underwent mechanical thrombectomy at The Affiliated Yongchuan Hospital of Chongqing Medical University and presented with hyperdense signs on their first postoperative CT scan. The patients were divided into a training set (*n* = 55, treated between August 2020 and March 2022) and a validation set (*n* = 41, treated between November 2022 and December 2023) for temporal validation of the predictive model. Of these, 61 patients (63.54%) were male and 35 (36.46%) were female, with ages ranging from 32 to 101 years (mean age: 66.38 ± 11.58 years). The enrolled patients were followed up and classified into a good prognosis group (mRS 0–2) and a poor prognosis group (mRS 3–6) based on the 90-day postoperative modified Rankin Scale (mRS) score, where a score of 6 indicated death. Inclusion criteria included: (i) Age ≥ 18 years; (ii) Confirmed large-artery occlusion based on computed tomography angiography (CTA), magnetic resonance angiography (MRA), or digital subtraction angiography (DSA). Exclusion criteria included: (i) Incomplete imaging or clinical data; (ii) Poor-quality imaging data; (iii) Absence of hyperdense signs on the first postoperative CT scan; (iv) History of intraoperative stent placement; (v) Loss to follow-up (Figure 1). This study was approved by the Ethics Committee of Yongchuan Hospital affiliated with Chongqing Medical University (No. 2024-41).

### 2.2 Information collection

Clinical and imaging data were collected, including age, gender, medical history, blood pressure, National Institutes of Health Stroke Scale (NIHSS) score, mRS score, volume of high-density areas, volume of low-density areas, and total volume of high-density and low-density areas, among others. Hyperdense signs were defined as hyperattenuating areas present at the thrombectomy site or distributed along the sulci and gyri. Hypodense signs were defined as hypoattenuating areas surrounding the hyperdense areas, with a density lower than that of the contralateral normal brain tissue. This study included patients who underwent mechanical thrombectomy for occlusion of the following major arteries: the middle cerebral artery (MCA), the internal carotid artery (ICA), the intracranial ICA, the intracranial vertebral artery, and the basilar artery.

The region of interest (ROI) was defined on the first postoperative non-contrast CT scan as any area surrounding the surgical cavity exhibiting density alterations relative to normal brain parenchyma. Delineation was performed semi-automatically using uAI-HematomaCare software. The resulting contours were independently reviewed slice by slice by two radiologists, who manually excluded obvious artifacts, pneumatosis within the surgical tract, and irrelevant calcifications. The following parameters were quantitatively assessed within the finalized ROI: (A) volume of the high-density areas (mL); (B) volume of the low-density areas (mL); (C) total abnormal volume (mL); and (D) mean CT value (HU) of the high-density region.

### 2.3 Statistical analysis

Statistical analysis was performed using SPSS 26.0 and R version 4.3.0 software. Continuous variables that followed a normal distribution were expressed as mean ± standard deviation (SD), and comparisons between the two groups were conducted using independent samples t-tests. Continuous variables that did not follow a normal distribution were described as median (interquartile range) [M(P_25_, P_75_)], and comparisons were made using the Mann–Whitney U test. Categorical variables were described using frequencies and percentages, and comparisons between groups were performed using the chi-square test. Univariate logistic regression analysis was conducted to identify statistically significant indicators, which were then included in multivariate logistic regression analysis. A *p*-value < 0.05 was considered statistically significant. The diagnostic performance of the model was evaluated using receiver operating characteristic (ROC) curves and the area under the curve (AUC). Internal validation of the model’s differentiation and calibration was performed using Bootstrap method with 500 resamples. The Hosmer–Lemeshow goodness-of-fit test was used to assess calibration, and decision curve analysis (DCA) was employed to evaluate net benefits.

## 3 Results

### 3.1 Patients characteristics

Among the 96 patients, 63 (65.6%) had MCA occlusion, 12 (12.5%) had internal carotid artery tandem middle cerebral artery occlusion, 8 (8.3%) had intracranial segment occlusion of the internal carotid artery, 5 (5.2%) had intracranial segment occlusion of the vertebral artery, and 8 (8.3%) had basilar artery occlusion. The good prognosis group comprised 46 patients, including 28 males and 18 females, with a mean age of 63.74 ± 10.78 years. The poor prognosis group comprised 50 patients, including 33 males and 17 females, with a mean age of 68.8 ± 11.86 years. Age, history of diabetes, admission diastolic blood pressure, preoperative NIHSS score, preoperative mRS score, D-dimer levels, volume of high-density areas, and total volume of high-density and low-density areas were significantly higher in the poor prognosis group compared to the good prognosis group (*p* < 0.05) (Table 1). Using the time period validation method, the enrolled patients were divided into a training group (*n* = 55) and a validation group (*n* = 41) in an approximate 1:1 ratio. No statistically significant differences were observed in clinical data between the two groups (Table 2).

**Table 1:** Differential analysis of good prognosis group and poor prognosis group.

Parameters	Good prognosis group (*n* = 46)	Poor prognosis group (*n* = 50)	*p*
Age (years)^*^	63.74 ± 10.78	68.80 ± 11.86	0.032
Sex (male/female)	28/18	33/17	0.602
Medical history			
Hypertension	26 (56.52)	29 (58.00)	0.884
Diabetes	5 (10.87)	9 (18.00)	0.035
Coronary heart disease	4 (8.70)	7 (14.00)	0.183
Atrial fibrillation	4 (8.70)	7 (14.00)	0.415
Smoking history	18 (39.13)	26 (52.00)	0.206
Drinking history	19 (41.30)	25 (50.00)	0.393
Blood platelet^†^	0.61 (0.30–1.72)	185.50 (149.50–232.75)	0.306
D-dimer^†^	0.61 (0.30–1.72)	2.05 (0.80–6.78)	<0.001
Admission diastolic blood pressure (mmHg)^*^	140.02 ± 19.17	152.80 ± 25.70	0.007
Admission systolic blood pressure (mmHg)^†^	82.00 (75.25–89.00)	85.00 (78.00–96.00)	0.226
Preoperative NIHSS score^†^	14.00 (9.00–17.00)	19.50 (14.00–22.75)	<0.001
Preoperative mRS score^†^	4.00 (4.00–4.75)	4.00 (4.00–5.00)	0.026
Time from onset to femoral artery puncture (h)^†^	7.00 (4.62–12.75)	7.00 (4.00–10.50)	0.535
mTICI grade (2b/3)	19/27	20/30	0.760
Lateral category (unilateral/bilateral)	42/4	41/9	0.183
Site of vascular occlusion (anterior cerebral circulation/posterior cerebral circulation)	45/1	38/12	0.002
First postoperative CT scan			
High density sign CT value (HU)^†^	49.35 (44.55–57.02)	51.95 (48.20–59.77)	0.072
Volume of high-density areas (mL)^†^	10.05 (3.32–24.60)	33.35 (13.18–63.65)	<0.001
Volume of low-density areas (ml)^†^	2.75 (0.00–13.60)	4.90 (0.00–34.12)	0.133
Total volume of high-density and low-density areas (ml)^†^	17.95 (6.22–34.23)	38.75 (16.52–91.78)	<0.001

^*^Data are means ± SDs, with ranges in parentheses.

^†^Data are medians, with IORs in parentheses.

**Table 2:** Comparison of clinical data between training and validation groups.

Parameters	Training group (*n* = 55)	Validation group (*n* = 41)	*p*
Age (years)^*^	66.15 ± 12.01	66.68 ± 11.11	0.823
Sex (male/female)	35/20	26/15	0.982
Medical history			
Hypertension	27 (49.09)	28 (68.29)	0.060
Diabetes	11 (20.00)	8 (19.51)	0.953
Coronary heart disease	5 (9.09)	8 (19.51)	0.140
Atrial fibrillation	3 (5.45)	8 (19.51)	0.070
Smoking history	27 (49.09)	17, 41.46	0.458
Drinking history	28 (50.91)	16, 39.02	0.248
Blood platelet^†^	202.00 (161.00–240.00)	172.00 (132.00–211.00)	0.057
D-dimer^†^	1.26 (0.44–4.48)	1.07 (0.40–2.28)	0.493
Admission diastolic blood pressure (mmHg)^*^	146.36 ± 22.22	147.10 ± 25.56	0.881
Admission systolic blood pressure (mmHg)^†^	84.00 (76.00–93.50)	81.00 (77.00–92.00)	0.447
Preoperative NIHSS score^†^	15.00 (10.00–21.00)	15.00 (11.00–19.00)	0.640
Preoperative mRS score^†^	4.00 (4.00–5.00)	4.00 (4.00–5.00)	0.874
Time from onset to femoral artery puncture (h)^†^	7.00 (4.00–18.00)	7.00 (4.00–11.00)	0.601
First postoperative CT scan			
High density sign CT value (HU)^†^	50.90 (46.30–57.05)	50.90 (46.60–59.60)	0.801
Volume of high-density areas (mL)^†^	12.70 (2.85–33.70)	24.30 (5.40–59.10)	0.093
Volume of low-density areas (mL)^†^	3.70 (0.00–19.15)	4.50 (0.00–24.40)	0.912
Total volume of high-density and low-density areas (mL)^†^	19.10 (5.40–43.32)	30.10 (9.70–72.10)	0.203

^*^Data are means ± SDs, with ranges in parentheses.

^†^Data are medians, with IORs in parentheses.

### 3.2 Risk factor analysis

Statistically significant indicators from the good and poor prognosis groups were included in univariate logistic regression analyses. The results revealed that age, history of diabetes, site of vascular occlusion, D-dimer, admission diastolic blood pressure, preoperative NIHSS score, volume of high-density areas, and total volume of high-density and low-density areas were significant factors associated with poor prognosis in patients with combined hyperdense signs (*p* < 0.05). Multifactorial logistic regression analysis further demonstrated that site of vascular occlusion, D-dimer, admission diastolic blood pressure, preoperative NIHSS score, volume of high-density areas, and total volume of high-density and low-density areas were independent risk factors for 90-day poor prognosis in patients with combined hyperdense signs (*p* < 0.05) (Table 3).

**Table 3:** Logistic regression analysis.

Parameters	Single factor					Multifactorial				
	*β*	SE	*Z*	*p*	OR (95%CI)	*β*	SE	*Z*	*p*	OR (95%CI)
Age (years)	0.04	0.02	2.09	0.036	1.04 (1.01–1.08)					
History of diabetes	−1.16	0.57	−2.04	0.041	0.31 (0.10–0.96)					
Site of vascular occlusion	2.65	1.06	2.49	0.013	14.21 (1.77–114.34)	2.59	1.22	2.13	0.033	13.28 (1.23–143.67)
D-dimer	0.26	0.09	2.87	0.004	1.30 (1.09–1.56)	0.26	0.11	2.48	0.013	1.30 (1.06–1.60)
Admission diastolic blood pressure (mmHg)	0.02	0.01	2.58	0.010	1.03 (1.01–1.04)	0.03	0.01	2.43	0.015	1.03 (1.01–1.06)
Preoperative NIHSS score	0.14	0.04	3.63	<0.001	1.15 (1.07–1.24)	0.12	0.06	2.13	0.033	1.13 (1.01–1.26)
Preoperative mRS score	0.44	0.24	1.80	0.071	1.55 (0.96–2.50)					
Volume of high-density areas (mL)	0.01	0.01	2.08	0.037	1.01 (1.01–1.02)	−0.05	0.03	−2.14	0.033	0.95 (0.90–0.99)
Total volume of high-density and low-density areas	0.02	0.01	2.86	0.004	1.02 (1.01–1.03)	0.06	0.02	3.03	0.002	1.07 (1.02–1.11)

OR, odds ratio; CI, confidence interval.

### 3.3 Model performance

**Figure 2:**
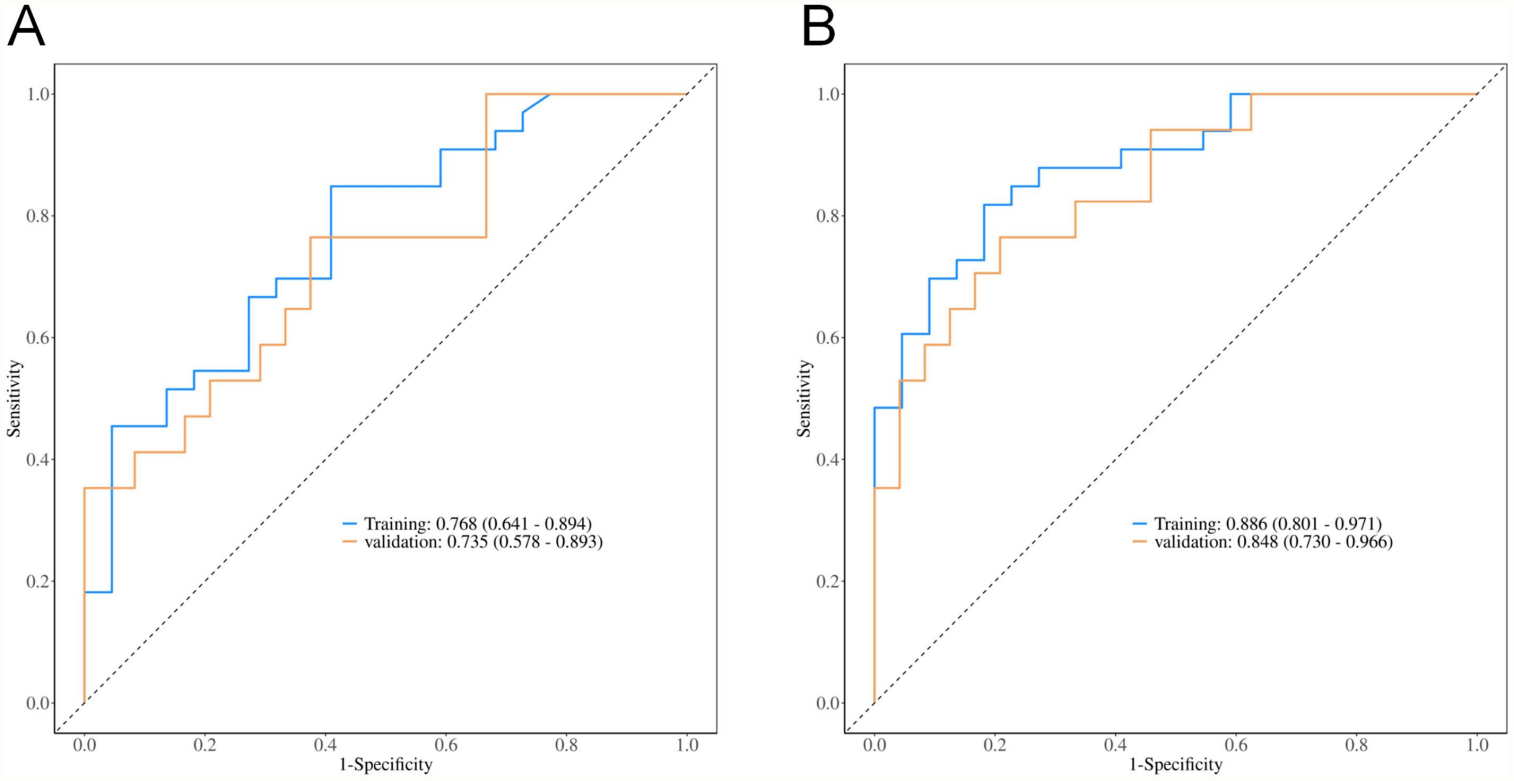
ROC curves. **(A)** Total volume; **(B)** Combined indicators.

ROC analysis demonstrated that the total volume of hyperdense and hypodense areas exhibited good predictive performance for 90-day poor prognosis in patients presenting with hyperdense signs on the first non-contrast CT scan after mechanical thrombectomy. The AUC was 0.768 (95% CI: 0.641–0.894) in the training set and 0.735 (95% CI: 0.578–0.893) in the validation set. A combined model incorporating vascular occlusion site, D-dimer level, admission diastolic blood pressure, preoperative NIHSS score, hyperdense volume, and total volume of hyperdense and hypodense areas achieved the highest predictive performance, with an AUC of 0.886 (95% CI: 0.801–0.971) in the training set and 0.848 (95% CI: 0.730–0.966) in the validation set (Table 4 and Figure 2).

**Table 4:** Predictive efficacy of the model.

Model	Formation	AUC (95% CI)	Accuracy	Sensitivity	Specificity	Cut-off value
Total volume of high-density and low-density zones	Training queue	0.768 (0.641–0.894)	0.745	0.591	0.848	10.9
	Validation queue	0.735 (0.578–0.893)	0.610	0.333	1.000	10.9
Combined indicators	Training queue	0.886 (0.801–0.971)	0.818	0.818	0.818	0.525
	Validation queue	0.848 (0.730–0.966)	0.707	0.542	0.941	0.525

**Figure 3:**
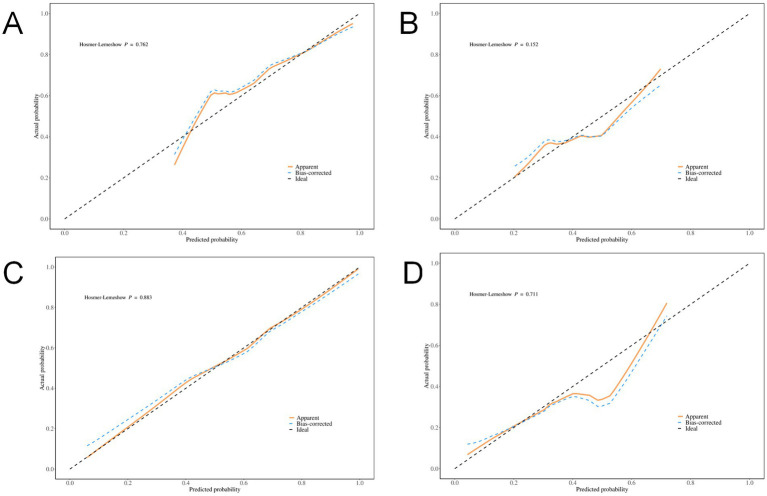
Calibration Curves. **(A)** Total volume of the training set; **(B)** Total volume of the validation set; **(C)** Combined diagnostic indicators of the training set; **(D)** Combined diagnostic indicators of the validation set.

Internal validation of the model’s discrimination and calibration was performed using the bootstrap method with 500 resamples. The model showed strong discriminative ability in predicting the risk of 90-day poor prognosis among patients with hyperdense signs (*p* < 0.001, 95% CI: 0.014–0.038). Calibration curve analysis indicated that the predicted probabilities of poor 90-day prognosis from both the combined model and the total volume model were well-aligned with actual outcomes in the training and validation sets (all *p*-values from the Hosmer–Lemeshow goodness-of-fit test > 0.05, indicating satisfactory calibration performance) (Figure 3).

**Figure 4:**
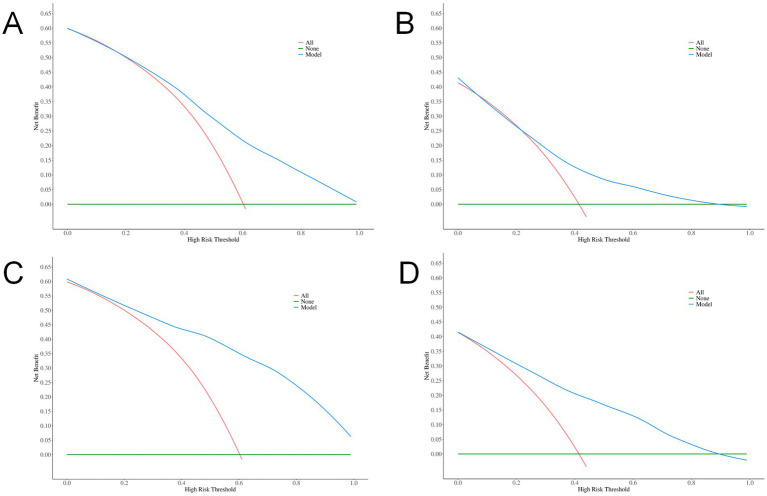
DCA curves. **(A)** Total volume in the training set; **(B)** Total volume in the validation set; **(C)** Combined indicators in the training set; **(D)** Combined indicators in the validation set.

DCA was conducted to evaluate the clinical utility of the models. The results revealed that both the total volume model (net benefit: 0.135 in the training set, 0.098 in the validation set) and the combined model (net benefit: 0.135 in the training set, 0.086 in the validation set) yielded positive net benefits across a wide range of threshold probabilities (10%–90%), supporting their potential value in clinical decision-making. Notably, the total volume model exhibited a broader range of threshold probabilities with positive net benefit (Figure 4).

## 4 Discussion

Some researchers have described the high-density areas on CT scan after mechanical thrombectomy as post-intervention cerebral hyperdensities (PCHDs) (13, 14), which may result from contrast staining, hemorrhagic transformation, or a combination of both. PCHDs are primarily associated with BBB disruption. Under normal conditions, the BBB permits the passage of only fat-soluble molecules with a molecular weight < 400 Da, while neither iodine-based contrast agents nor erythrocytes can penetrate it (15, 16). BBB dysfunction can be induced by injury, ischemia, hypoxia, infection, toxins, and other pathological conditions (17, 18). When the damage is confined to vascular endothelial cells, only contrast agents extravasate from the blood vessels. However, when the basement membrane is compromised, both blood components and/or contrast agents may extravasate, potentially leading to hemorrhagic transformation (19). Previous studies have demonstrated that patients with acute ischemic stroke who undergo mechanical thrombectomy and exhibit high-density CT signs at 24-h follow-up are at increased risk of higher mortality and poorer prognosis (4, 5, 10). Differentiating between postoperative cerebral hemorrhage and contrast extravasation based on conventional CT scans is challenging, as both present as high-density areas. When the CT value exceeds 90 Hounsfield Units (HU), it is often assumed that contrast staining is present, However, the possibility of hemorrhage cannot be entirely excluded. Previous research has predominantly concentrated on the predictive value of hemorrhage and prognosis based on CT scans, emphasizing on the presence of high-density signs and imaging characteristics of hyperdense lesions (including CT values, distribution, shape, and the presence of mass effects). However, few studies have quantitatively assessed changes within and surrounding high-density areas. In this study, volume of high-density areas and total volume of high-density and low-density areas were higher in the poor prognosis group compared to the good prognosis group. The total volume of high-density and low-density areas demonstrated high specificity (75.8%) in predicting poor prognosis. A higher volume of high-density areas may indicate extensive extravasation of contrast agents and blood components, indirectly reflecting the severity of vascular injury (20). Contrast agents are neurotoxic, and the extravasation of blood components can lead to insufficient reperfusion of surrounding brain tissue and exacerbate inflammatory responses (21). These factors increase the risk of hemorrhage following thrombectomy and worsen cerebral edema. The combined effects of these pathological processes progressively exacerbate brain tissue damage, which explains why patients with larger total volumes of high-density and low-density areas in this study face a higher risk of poor prognosis. In addition, studies have shown that elevated blood pressure, longer duration from symptom onset, higher preoperative NIHSS scores, and larger cerebral infarct volumes further exacerbate poor outcomes (22–25). In this study, age and preoperative NHISS score were higher in the poor prognosis group compared to the good prognosis group. The American Stroke Association (ASA) notes that elderly patients often exhibit lower vital signs and reduced tolerance to treatment compared to younger patients, leading to a higher likelihood of poor prognosis (26). This observation is largely consistent with the findings of this study. Guidelines indicate that 3-month all-cause mortality is significantly associated with higher or lower baseline blood pressure in patients undergoing endovascular therapy (1). In this study, admission diastolic blood pressure was significantly higher in the poor prognosis group compared to the good prognosis group, a finding consistent with Fu et al.’s (27) study on risk factors for 90-day mortality after endovascular treatment of ischemic stroke in elderly patients. This may be attributed to the fact that diastolic blood pressure reflects the reserve capacity of the peripheral vasculature, and elevated diastolic blood pressure can impair the reestablishment of blood flow following reperfusion.

This study has several limitations: (i) As a single-center retrospective study with a relatively small sample size, selection bias may be unavoidable. (ii) Due to the limited number of enrolled cases, the analysis did not explore potential differences in influencing factors between patients with anterior and posterior circulation strokes. (iii) Lack of longitudinal follow-up of patients. In conclusion, the total volume of high-density and low-density areas is an independent predictor of poor prognosis. Quantitative assessment of postoperative CT scans provides valuable insights for guiding clinical decision-making and improving patient outcomes.

## Data Availability

The original contributions presented in the study are included in the article/supplementary material, further inquiries can be directed to the corresponding author.
